# Microvesicle delivery of a lysosomal transport protein to ex vivo rabbit cornea

**DOI:** 10.1016/j.ymgmr.2020.100587

**Published:** 2020-04-07

**Authors:** Jess G. Thoene, Monte A. DelMonte, Jodi Mullet

**Affiliations:** a Department of Pediatrics, Division of Pediatric Genetics, Metabolism and Genomic Medicine, University of Michigan, Ann Arbor, MI 48109, USA; b Department of Ophthalmology and Visual Sciences, Division of Pediatric Ophthalmology, Kellogg Eye Center, University of Michigan, Ann Arbor, MI 48105, USA

**Keywords:** Cystinosin, Microvesicles, Cornea, Rabbit, Therapy

## Abstract

Therapeutic use of transmembrane proteins is limited because of irreversible denaturation when away from their native lipid membrane. Mutations in lysosomal membrane transport proteins cause many lethal disorders including cystinosis which results from mutations in CTNS, which codes for the lysosomal cystine transport protein, cystinosin. Cystinosin-deficient fibroblasts, including keratocytes (corneal fibroblasts) accumulate lysosomal cystine. Cystinosis patients develop highly painful corneal cystine crystals, resulting in severe visually debilitating photophobia. The only available therapy is daily treatment with cysteamine eye drops. We have previously shown that microvesicles containing functional cystinosin are spontaneously produced by infecting *Spodoptera frugiperda* cells (Sf9) with baculovirus containing human wt CTNS. Infecting Sf9 cells for 3 days at a MOI of 1 yields 10^11^microvesicles /ml with a modal diameter of 90 nm. Addition of these vesicles to cultures of cystinotic fibroblasts produces cystine depletion over the course of 96 h, which persists for 2 weeks. In this paper we show that addition of such microvesicles containing cystinosinGFP to ex vivo rabbit ocular globes yields punctate perinuclear green fluorescence in the corneal keratocytes. These results support potential therapeutic use of these cystinosin containing microvesicles in treating cystinotic corneal keratopathy with the advantage of administering twice/month instead of daily topical administration.

## 1 Introduction

Cystinosis is an inborn error of lysosomal cystine transport caused by a defect in the lysosomal transmembrane cystine transport protein, cystinosin [1]. This defect in transmembrane transport results in the lysosomal accumulation of the disulfide aminoacid cystine [2]. The principal clinical manifestations of cystinosis are: a) failure of tubular reabsorption of small molecules including electrolytes and water (renal Fanconi Syndrome), b) progressive renal failure [2], c) failure to thrive [2], d) muscle wasting in the third decade of life [3], e)CNS abnormalities [4], f) endocrine complications [5], g) pulmonary and bone complications [6,7], h) retinopathy, and i) corneal stromal cystine crystal accumulation, resulting in severe photophobia and eye pain which may progress to corneal opacity/scarring and blindness [8]. Currently the keratopathy of cystinosis is treated with a topical cysteamine solution, Cystaran•, or Cystadrops• (cysteamine HCL 0.44%, or 0.37%, respectively) [9], which effectively eliminates the corneal cystine crystals. The eye drops must be initially administered at least four to six times per day, which hinders adequate compliance, particularly in younger patients. A new, viscous form, which may be administered as few as 4 times per day has been described [10]. A recent study in cystinosin knock out mice using cysteamine-containing nanowafers has shown promise in reducing the drug administration frequency to once per day. The material, however, must be administered to the cornea with a fingertip [11].

In this paper we describe a new protein delivery system which employs microvesicles containing human wild type cystinosin obtained by infection of cultured Spodoptera cells with Baculovirus containing the human gene for cystinosin (CTNS) tagged with GFP. We have previously demonstrated the presence of human wild type cystinosin in the vesicles by LC/MS/MS and have shown bioactivity via cystine depletion of cultured cystinotic fibroblasts.

Such treatment of cystinotic fibroblasts produced a decrease in cell cystine content in 96 h from 3.0 ± 0.09 nmol/mg protein to 1.3 ± 0.5 nmol/mg protein (*p* = .0001), whereas control cells incubated for 96 h in media lacking microvesicles remained at 2.8 ± 0.7 nmol/mg protein after 96 h [12]. We now demonstrate microvesicle-mediated entry of cystinosinGFP into ex vivo rabbit cornea with penetration in 96 h to approximately one half of the corneal depth.

## 2 Materials and methods

### 2.1 Microvesicle preparation and characterization

Microvesicles were prepared and characterized as previously described [12]. Briefly, *Spodoptera frugiperda* (Sf9) cells were cultured for 4 days in suspension in SF900 II insect culture media @ 27 °C and infected with Baculovirus containing the hCTNS-GFP sequence with an MOI of 1.0. This was performed under contract by the University of Iowa Viral Vector Core. The four day post-infection supernatant from the Sf9 cells was sent frozen on dry ice. Upon receipt the material was thawed, dialyzed (MW cutoff 3500) into Ham s F12 with Pen, Strep and Fungizone, but not FCS for 48 h at a final dilution of 1:2500, and then sterilized via 0.22 μ filtration and stored @ 4C° until used. The vesicles so produced retain cystine-depleting activity for 4 weeks at 4 °C, and 6 months at −80 °C.

Microvesicles were counted and sized using a NanoSight instrument at the Malvern NanoSight Core at the University of Michigan. Each batch of microvesicles was assessed for bioactivity by cystine depletion of cystinotic fibroblasts produced by addition of 10^11^ vesicles/ml to cultured human cystinotic fibroblasts (GM0008) incubated at 37 °C, in modified Hams F12 media with pen, strep and fungizone in a humidified CO2 (5%) flushed incubator, and followed by fibroblast harvest at 4 days for subsequent shipment to the Cystine Determination Laboratory at UCSD for cystine measurement. Cystine depletion of fibroblasts reached or exceeded 50% at 96 h as previously reported [12]. Protein LC-MS/MS was performed by the University of Michigan Proteomics & Peptide Synthesis Core.

### 2.2 Fibroblast and ocular globe studies

#### 2.2.1 Fibroblasts

Fibroblasts were cultured for 96 h in Ham's F12 as described in Sec 2.1, exposed to control medium lacking microvesicles, or to medium containing10^11^ cystinosinGFP microvesicles/ml for 96 h, then processed and prepared for fluorescent microscopy as described in Sec 2.3.1.

#### 2.2.2 Rabbit ocular globes

Rabbit globes were obtained post mortem after euthanasia from normal wild type NZW animals at the University of Michigan, removed immediately post-mortem, washed three times in sterile PBS, and transported to the tissue culture laboratory. The animals were humanely euthanized for other experiments not involving the ocular globes, and approved by the University of Michigan Institutional Animal Care and Use Committee, protocol #PRO00008218. All animal work was carried out in accordance with relevant guidelines and legislation at the University of Michigan. Individual intact globes were incubated at 37C in a 5%CO2 incubator in 150 ml flasks with gas-permeable stoppers containing 50 ml Ham s F12 medium supplemented with or without the addition of 10^11^/ml cystinosinGFP-containing microvesicles.

At the times indicated, the globes were removed, washed 3 times in sterile PBS with Vortex agitation, and the cornea dissected from the globe at the cornea-scleral limbus, and processed as described in section 2.3. Human cadaver ocular globes for corneal transplantation are stored in tissue culture media for up to 28 days with preservation of function and corneal viability [13,14].

### 2.3 Microscopy

#### 2.3.1 Fibroblasts

Following harvest described above, the cultured fibroblasts were pelleted in a microcentrifuge, the pellet then immediately transferred to 4% paraformaldehyde for subsequent sectioning and viewing.

#### 2.3.2 Intact cornea preparation

Corneas obtained as described in section 2.2.2 were imbedded in Histogel/OCT at the University of Michigan Microscopy and Image Analysis Laboratory (MIL) and 20 μ sections prepared, mounted in Molecular Probes DAPI Diamond mounting media, and viewed for green fluorescence with a Nikon inverted confocal fluorescent microscope. Prior to examining treated cells the background was set to eliminate the intrinsic green fluorescence seen in control cells and cornea,.

#### 2.3.3 Scanning electron microscopy of fibroblasts

After 96 h incubation with vesicles, cells were immediately fixed in 3% paraformaldehyde and 2.5% glutaraldehyde in 0.1 M Sorensen's buffer. They were then washed in Sorenson's buffer with two additional changes of buffer before osmium fixation and imaging.

## 3 Results

### 3.1 Microvesicle characteristics

As previously reported [12], the density on ultracentrifugation is 1.05 *g*/ml, which is consistent with high lipid content, since the density of proteins is 1.22–1.43 g/ml [15], hence they are relatively protein-poor and lipid rich. They are LBPA negative, the modal vesicle size is ~100 nm and the concentration of microvesicles in growth media after the lytic phase is ~10^11^/ml (Supplemental Fig. 1). The microvesicles display lipid bilayer membrane structure on transmission electron microscopy (Supplemental Fig. 2) [12].

### 3.2 Proteomic analysis of microvesicles

LC/MS/MS spectrometry of the vesicles performed by the University of Michigan Proteomics and Peptide Synthesis Core identified a number of insect proteins using the Insecta database (Supplemental Table 1), including moesin, a member of the ERM family of proteins which serve to cross-link cytoskeleton and plasma membrane [16]. Human cystinosin was specifically identified in the baculovirus-infected insect vesicles (Supplemental Table 2).

### 3.3 Microvesicle-mediated entry of cystinosin into ex vivo rabbit cornea

**Fig. 1:**
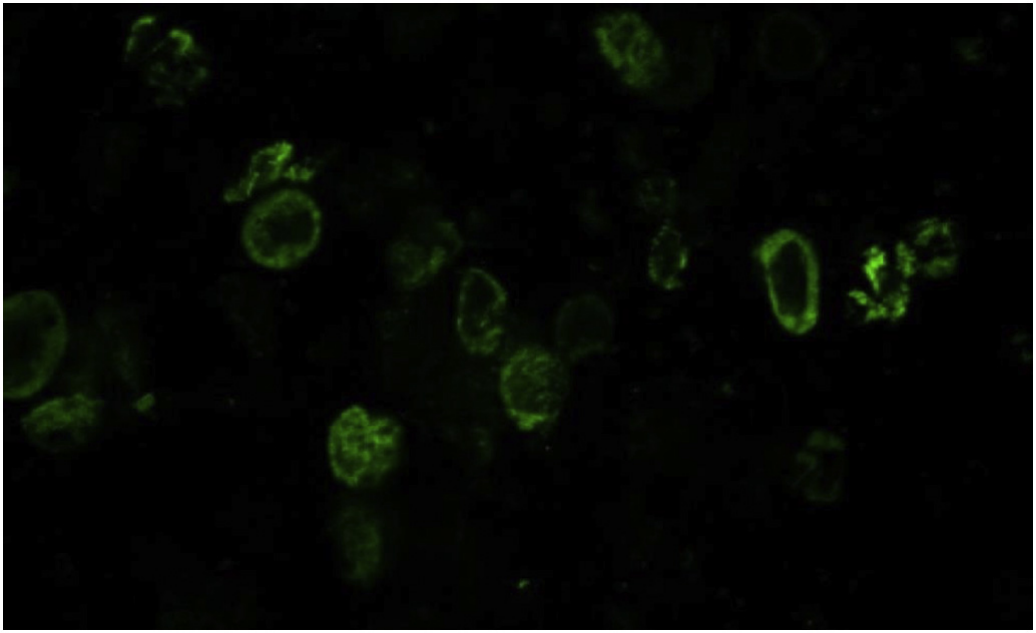
Fluorescent micrograph of Sf9 cells after infection with CtnsGFP Bac showing expression of GFP.

Infection of host Sf9 cells with baculovirus containing the human CTNSGFP sequence causes the appearance of GFP signal in the Sf9 cells (Fig. 1).

**Fig. 2:**
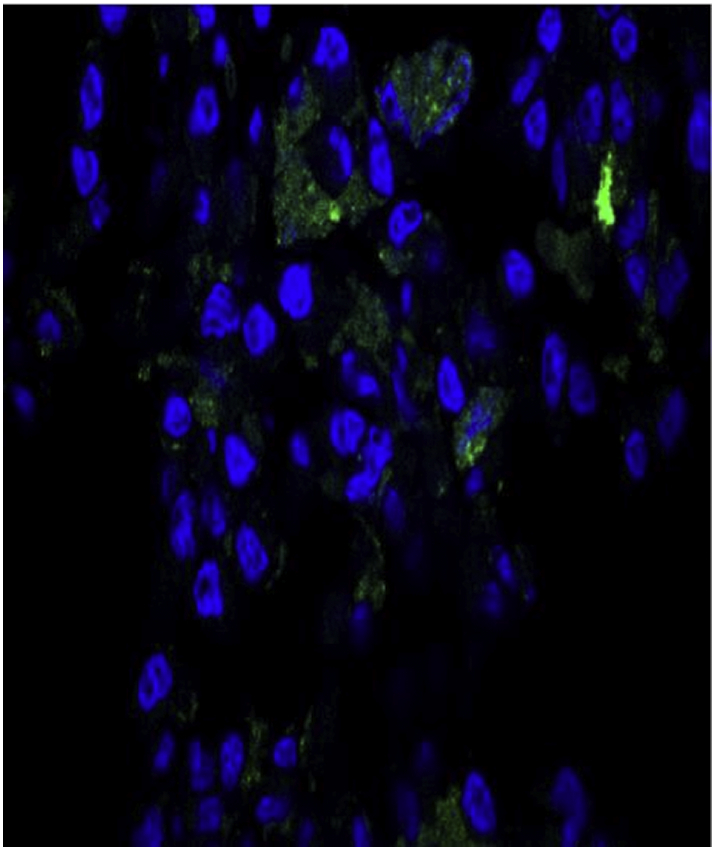
GFP fluorescence in cystinotic fibroblast line GM00090 after 96 h exposure to 10 ^11^ cystinosin-GFP microvesicles/ml showing presence of GFP signal transferred to the cultured fibroblasts by the cystinosinGFP microvesicles, Nuclei stained with DAPI. Fibroblast dimensions ~10 μ.

Addition of the microvesicles harvested as described from the infected Sf9 cells to cultured cystinotic human fibroblasts yields GFP signal in the fibroblasts in a perinuclear, cytoplasmic distribution (Fig. 2).

**Fig. 3:**
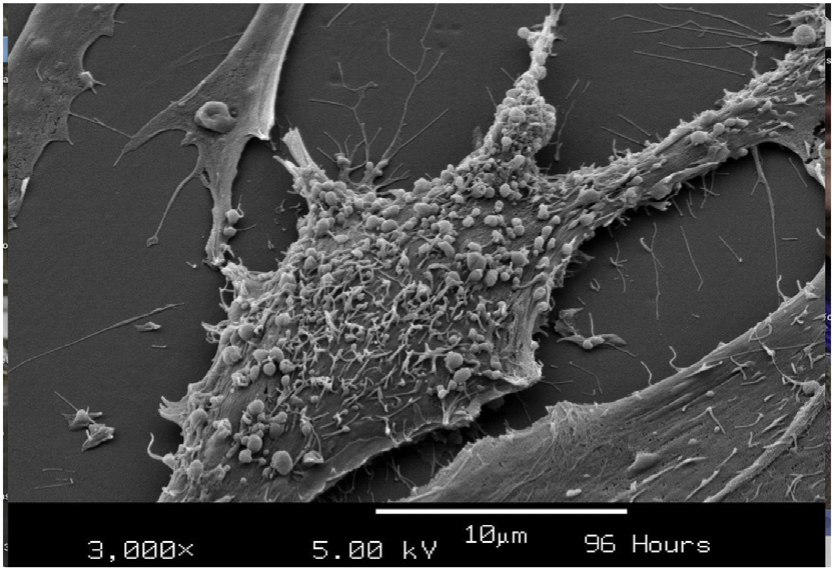
Scanning electron micrograph of human fibroblasts in tissue culture 96 h after exposure to 10^11^ cystinosin-containing microvesicles /ml. Multiple microvesicles are attached to the fibroblast plasma membrane.

Addition of 10^11^ microvesicles/ml for 96 h to cultured cystinotic fibroblasts, followed by harvest and examination by scanning electron microscopy displays abundant microvesicles attached to the plasma membrane of the cells (Fig. 3 and [12]).

**Fig. 4:**
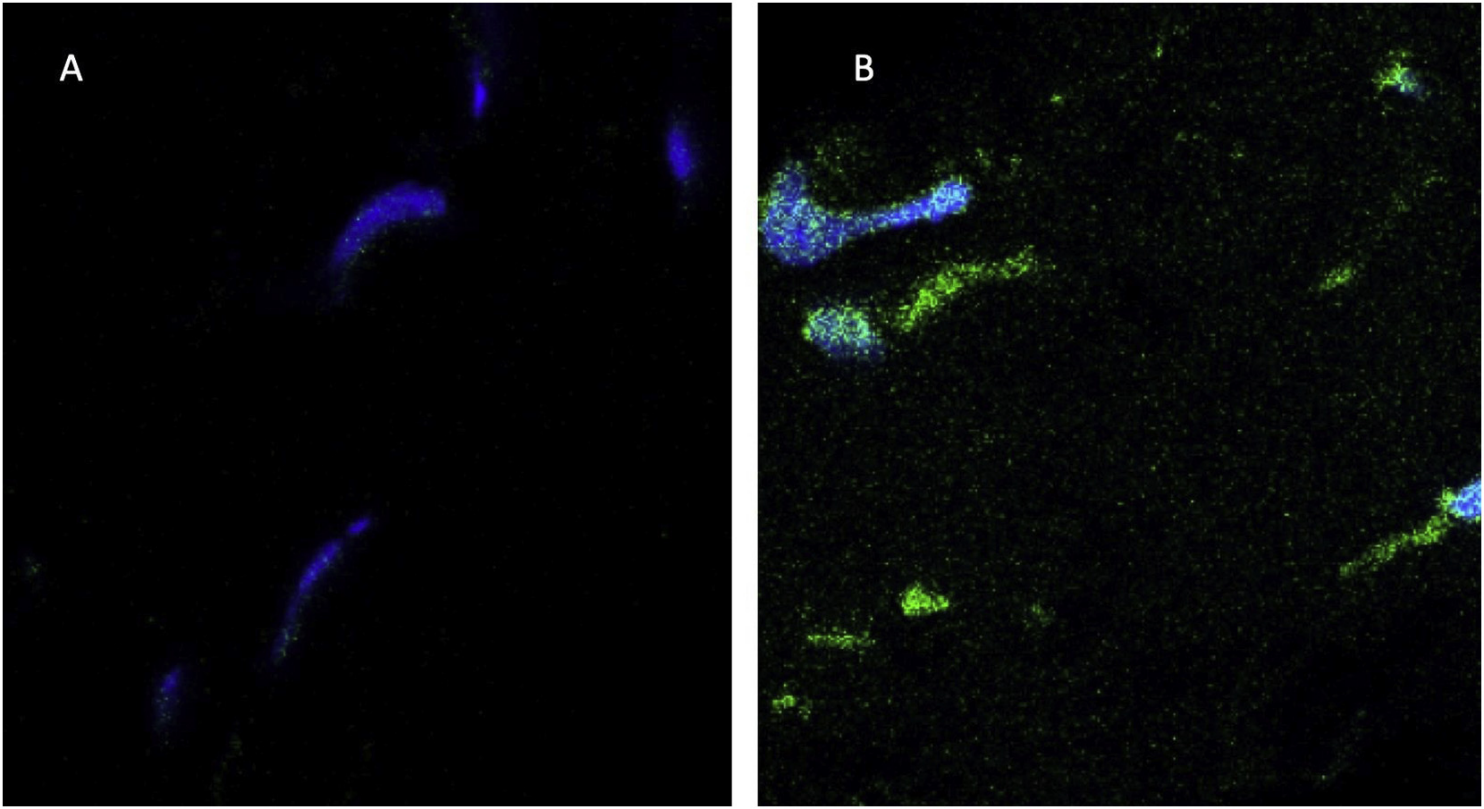
Delivery of CystinosinGFP ex vivo to Rabbit Ocular Globe. (A) Transverse corneal section of control without incubation with microvesicles. (B) Transverse section of cystinosinGFP-treated cornea after 96 h incubation showing multiple GFP-positive signals. Primary magnification 60×.

Incubation of ex vivo rabbit ocular globes without microvesicles yields no GFP fluorescence (Fig. 4A). Incubation of intact ex vivo rabbit ocular globes with 10^11^cystinosinGFP microvesicles/ ml produces extensive perinuclear green fluorescence at 96 h (Fig. 4B).

**Fig. 5:**
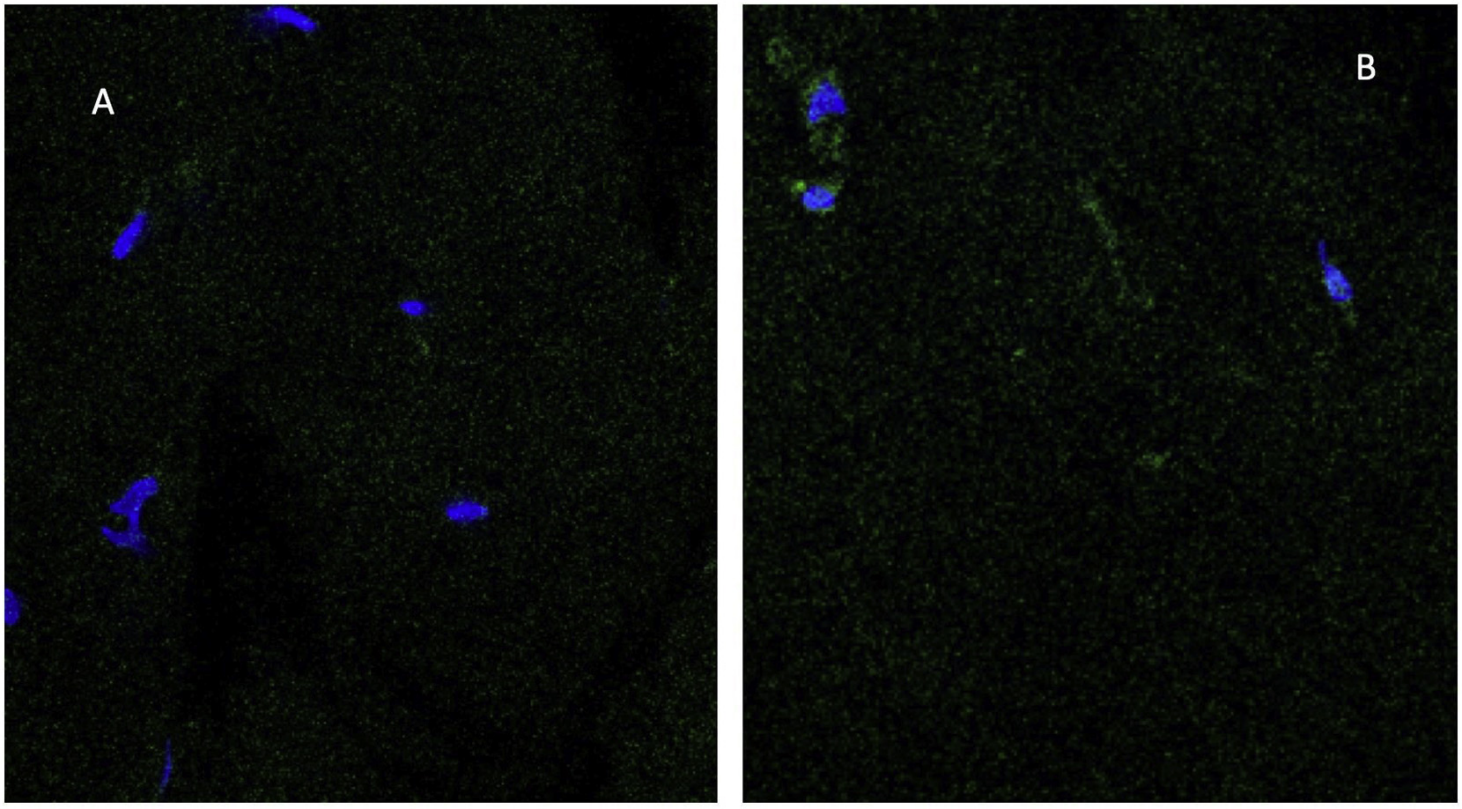
Time-dependence of expression of GFP in vesicle-treated cornea: After 48 h there is some GFP signal detectable (A), but after 96 h exposure there is prominent GFP signal (B), and as seen in the prior 96 h exposed cornea, Fig. 4B. (60 x magnification).

Delivery to ex vivo cornea is time dependent. After 48 h incubation of the rabbit ocular globe with cystinosinGFP microvesicles there is limited green fluorescence as shown in Fig. 5A. After 96 h incubation there is pronounced diffuse green fluorescence (Fig. 5B).

**Fig. 6:**
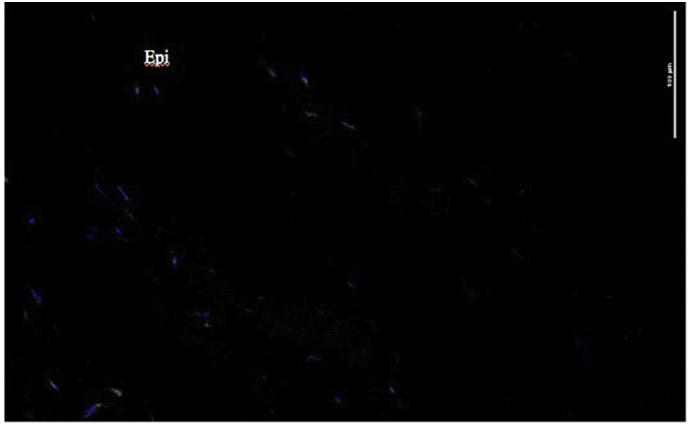
Penetration of GFP-cystinosin into rabbit cornea after 96 h incubation with cystinosinGFP vesicles. Size bar = 100 μ. Epi is epithelial surface of cornea. Average cornea thickness is ~500 μ.

A low power view of the treated cornea after 96 h incubation showed GFP signal reaching to about 50% of the 400 μ cornea thickness (Fig. 6).

## 4 Discussion

Baculovirus is a natural pathogen of Lepidoptera [12], causing a lytic infection after extensive virus replication in the host tissue. This finding led to developing Baculovirus as a vector for expressing many soluble proteins in vitro. The advantages are several including a host range limited to members of the class Insecta, and hence classified as BSL1 since it cannot replicate in mammalian cells. Further, the widely used host cells, *Spodoptera frugiperda*, grow in suspension at room temperature, facilitating trans protein production. This method was not thought feasible for production of membrane proteins, since such proteins irreversibly denature when removed from their native lipid bilayer environment [17]. However we discovered [12] that as a consequence of the lytic infection, membrane proteins cloned in Spodotera via Baculovirus are released in microvesicles and function to cause depletion of stored lysosomal cystine when vesicles containing the lysosomal transport protein cystinosin are added to cultured cystinotic fibroblasts [12].

Cystinosis keratopathy is currently treated by topical administration of cysteamine eye drops (Cystaran• and Cystadrops•) but requires lifelong frequent administration for maximum cystine crystal dissolution to occur, which is burdensome for parents and child alike. There is potential to alleviate this dosing problem using baculovirus/Spodoptera microvesicles containing the lysosomal cystine transport protein, cystinosin, as here described. Since cystine depletion in fibroblasts treated with these vesicles persists for up to 2 weeks [12], the frequency of ocular administration could be greatly diminished. Additionally, cystinosin is now thought to have significant functions besides merely transporting cystine from lysosomes [[18], [19], [20]], and failure of these functions may contribute to the keratopathy. Introduction of human wild-type cystinosin would be expected to address these issues as well as restoring cystine transport to lysosomes.

Microvesicles are an emerging area of cell biology which focused initially on diagnostic use. Currently microvesicles are increasingly recognized as a potential means to convey proteins [21,22]. Delivery of a therapeutic protein to the corneal stroma of cystinotic patients, a goal of treatment in many corneal disorders, is inhibited by the physicochemical barrier of Bowman's membrane which might be overcome by using this microvesicle delivery system. It may appear unlikely that microvesicles derived from insect cells could accomplish delivery of a human transport protein to the interior of cultured mammalian cells, however the basic lipid composition of biomembranes is conserved from the prokaryote/eukaryote divide [23] hence membrane fusion between insect and mammalian cells may not be surprising. Spodoptera cystinosin is homologous with that of other insects, including Bombyx and Drosophila. Spodptera Ctns is 74% homologous with CTNS [12]. Thus the fusion of these microvesicles with human fibroblast plasma membranes and delivery of cystinosin may not be hindered by structural constraints. Animal studies would therefore support clinical trials if preclinical trials are successful.

## Appendix A Supplementary data

Supplementary material Image 1
