# Supplementary material for: Microvesicle delivery of a lysosomal transport protein to ex vivo rabbit cornea
Source: Mol Genet Metab Rep. 2020 Apr 7;23:100587. doi: 10.1016/j.ymgmr.2020.100587 (PMC7138922; doi:10.1016/j.ymgmr.2020.100587)
Supplement: Supplementary file 1 — Supplementary material [file mmc1.docx]

**8.0 Supplemental Figures and Tables :**

.


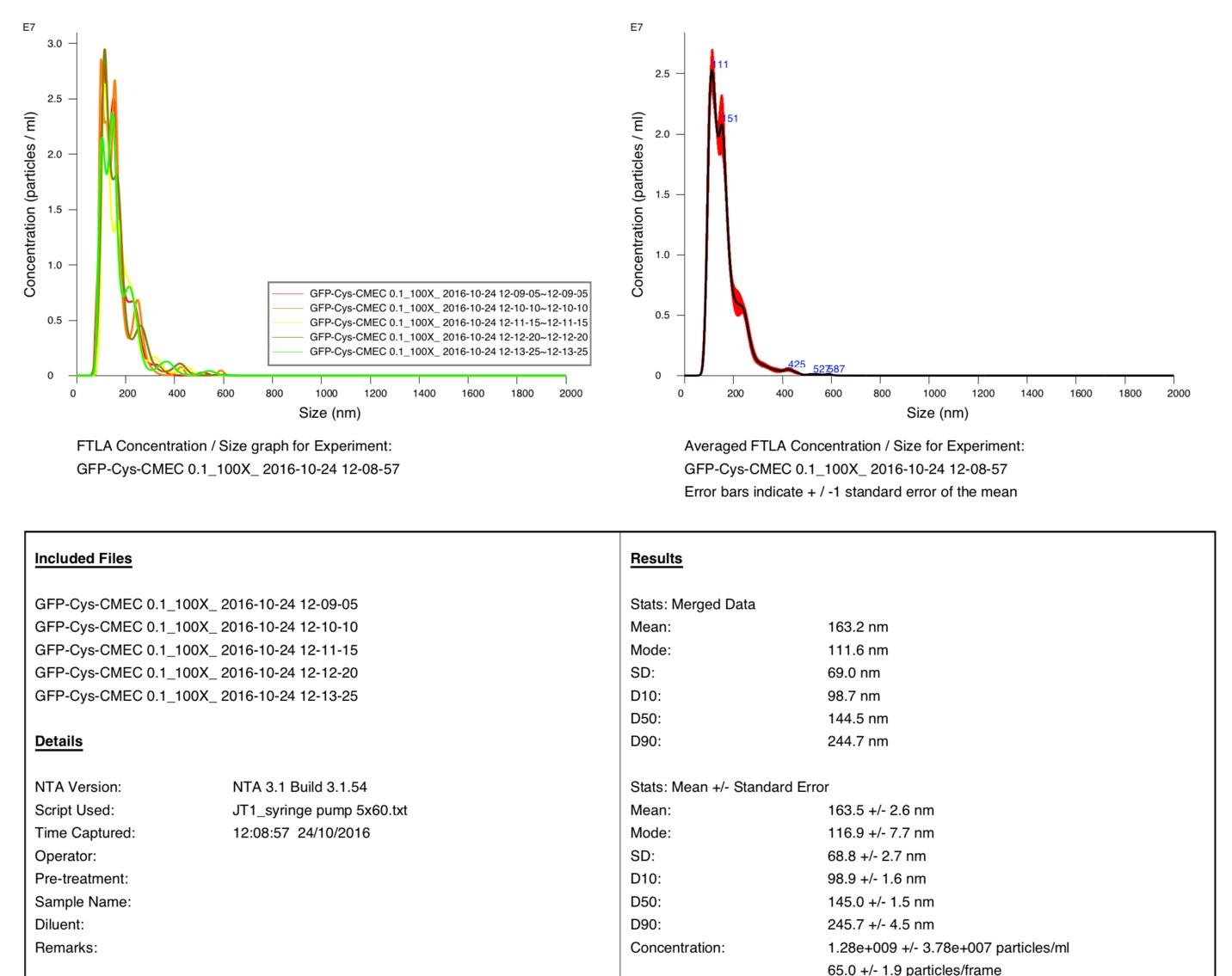


Supplemental Fig1: NanoSight analysis of vesicles showing ~100μ diameter and concentration of 109 at 1/100 dilution


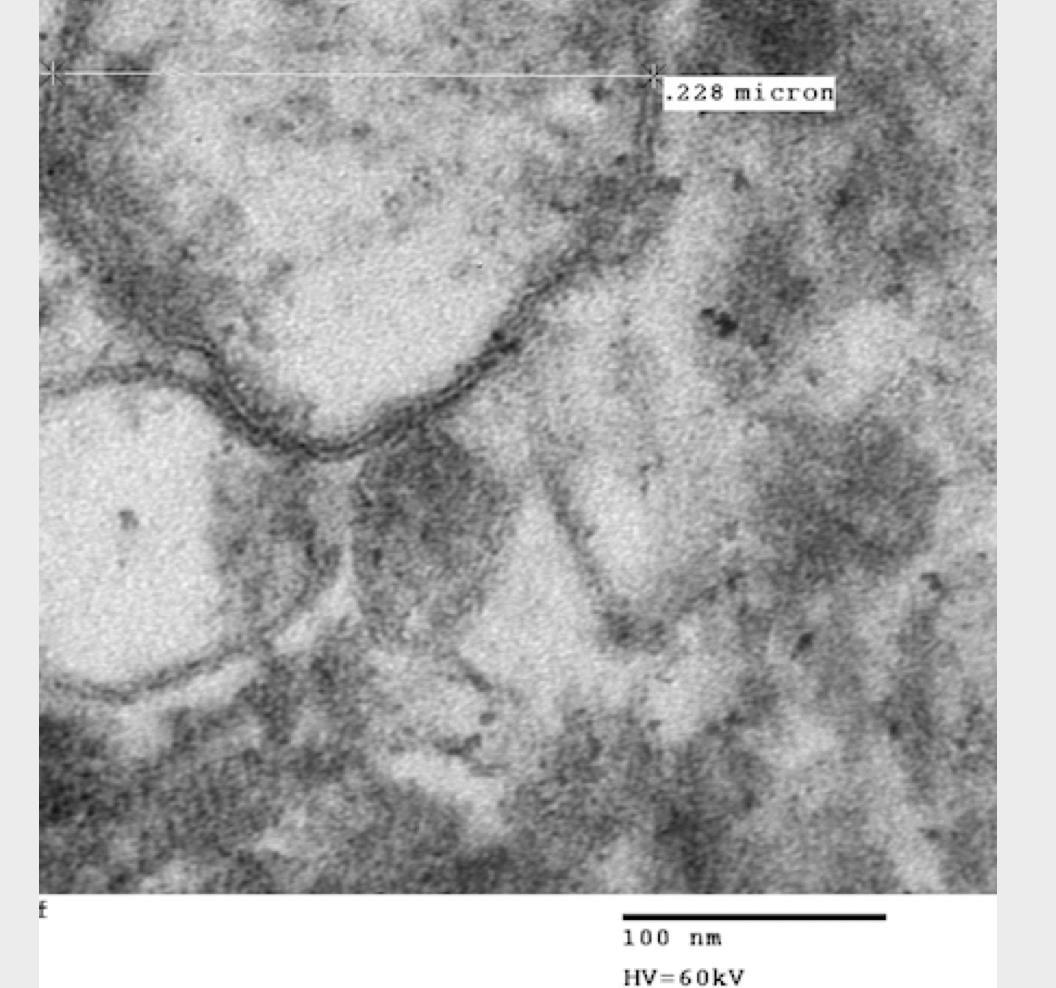
 Supplemental Fig. 2: Transmission electron micrograph of microvesicles displaying lipid bilayer structure.

Supplemental Table 1: Major proteins and source organism identified by LC/MS/MS/ in cystinosin-containing Spodoptera microvesicles

**beta-tubulin [Bombyx mori]**

**heat shock protein 90 [Spodoptera litura]
tubulin beta-1 chain [Pediculus humanus corporis]**

**alpha-tubulin [Xestia c-nigrum]**

**clathrin [Bombyx mori]**

**tubulin alpha-1 chain**

**transitional endoplasmic reticulum ATPase TER94 [Bombyx mori]**

**beta actin [Xestia c-nigrum]
actin 5 [Aedes aegypti]
polyubiquitin [Bombyx mori]**

**ribosomal protein S27A [Spodoptera frugiperda]**

**moesin [Spodoptera frugiperda]
dynein heavy chain [Tribolium castaneum]**

Supplemental Table 2

MSYYHHHHHHDYDIPTTENLYFQGAMDPEFMIRN WLTIFILFPLKLVEK**C ESSVSLTVPPVVK**LENGSSTNVSLTLRPPLNATLVITF EITFRSKNITIL

ELPDEVVVPPGVTNSSFQVTSQNVGQLTVYLHGNH SNQTGPRIR**FLVIR**S SAISIINQVIGWIYFVAWSISFYPQVIMNWRRKSVIG LSFDFVALNLTGF

VAYSVFNIGLLWVPYIK**EQFLLK**YPNGVNPVNSNDV FFSLHAVVLTLIII VQCCLYERGGQRVSWPAIGFLVLAWLFAFVTMIVA AVGVITWLQFLFCFS

YIKLAVTLVKYFPQAYMNFYYKSTEGWSIGNVLLDFT GGSFSLLQMFLQS

YNNDQWTLIFGDPTKFGLGVFSIVFDVVFFIQHFCL YRKR PGYDQLN

Peptides (bold) identified by LC-MS/MS in the human cystinosin sequence (including the His tag and linker) in microvesicles from Sf9 cells infected with BV containing the human CTNS sequence. (12)
